# Potential Therapeutic Drugs for Parkinson's Disease Based on Data Mining and Bioinformatics Analysis

**DOI:** 10.1155/2018/3464578

**Published:** 2018-10-02

**Authors:** Chuan Xu, Jiajun Chen, Xia Xu, Yingyu Zhang, Jia Li

**Affiliations:** ^1^Department of Neurology (III), China-Japan Union Hospital of Jilin University, Changchun 130033, Jilin, China; ^2^Yiwu Maternal and Child Health Hospital, Yiwu 322000, Zhejiang, China

## Abstract

The objective is to search potential therapeutic drugs for Parkinson's disease based on data mining and bioinformatics analysis and providing new ideas for research studies on “new application of conventional drugs.” Method differential gene candidates were obtained based on data mining of genes of PD brain tissue, original gene data analysis, differential gene crossover, pathway enrichment analysis, and protein interaction, and potential therapeutic drugs for Parkinson's disease were obtained through drug-gene relationship. *Result*. 250 common differential genes were obtained from 3 research studies, and 31 differential gene candidates were obtained through gene enrichment analysis and protein interaction. 10 drugs such as metformin hydrochloride were directly or indirectly correlated to differential gene candidates. *Conclusion*. Potential therapeutic drugs that may be used for prevention and treatment of Parkinson's disease were discovered through data mining and bioinformatics analysis, which provided new ideas for research and development of drugs. Results showed that metformin hydrochloride and other drugs had certain therapeutical effect on Parkinson's disease, and melbine (DMBG) can be used for treatment of Parkinson's disease and type 2 diabetes patients.

## 1. Introduction

With a high morbidity and a high disability rate, Parkinson's disease is the second degenerative disease of nervous system. Presently, treatment Parkinson's disease focuses on symptomatic treatment, which can just relief the symptoms and can neither effectively inhibit the progression of the disease nor cure it [1, 2]. Research studies on “new application of conventional drugs” based on differential genes of the brain tissue may enable to cure Parkinson's disease (PD). Aspirin is a famous drug with “new application of conventional drugs.” Aspirin was first applied to antipyretic-analgesic and anti-inflammatory treatment as a nonsteroidal anti-inflammatory drug. Then, it was found to be able to inhibit antiplatelet aggregation of TXA2, and thus it has been extensively applied to treatment of cardiovascular and cerebrovascular diseases [3, 4]. However, the mining model of indications for drug therapy was different from traditional drug R&D modes. The latter depended on physical tests, such as cell test, animal test, and clinical test, which were made to determine chemical components of relevant substances, and synthesis of drug compounds and featured high investment, high risk, and long R&D cycle [5, 6]. It was an alternative solution for drug R&D to readjust existing drugs for treatment of other diseases, guarantee of drug safety, lower cost, and higher R&D efficiency [7].

This paper was aimed at providing new drug candidates for PD treatment and providing new methods and ideas for drug screening through data mining and bioinformatics analysis of PD brain tissue.

## 2. Method

**Figure 1:**
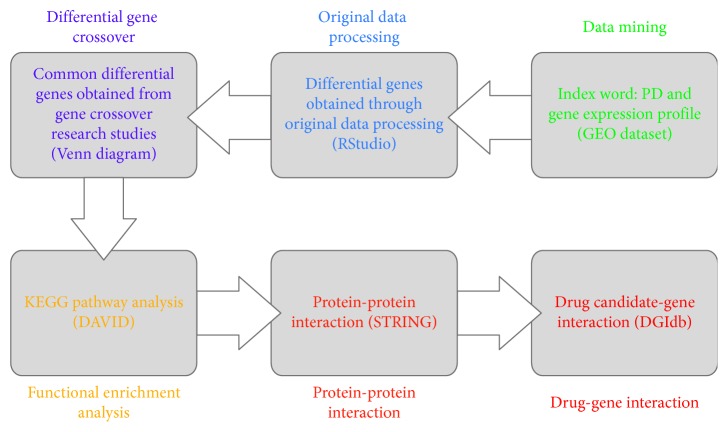
Technical route of data mining.

- As shown in Figure 1, “PD (Parkinson's disease)” and “gene expression profiling” were retrieved in GEO dataset (gene expression omnibus dataset), and references were screened. Selection criteria for retrieved references: the approval of Ethics Committee was indicated in the research; diagnosis of PD was demonstrated by clinic and neuropathology; the brain tissue of normal people and PD patients was the research object; original gene data can be obtained; original gene chip had high quality. Exclusion criteria: the approval of Ethics Committee was not indicated in the research; diagnosis of PD was not demonstrated by clinic and neuropathology; the research object was not the brain tissue of normal people and PD patients; original gene data cannot be obtained; original gene chip had poor quality. Screened original gene data was obtained and downloaded.
- The quality of original gene data was evaluated using R language and RStudio software, and logFC > 1 or logFC < (−1) was set to obtain differential genes.
- Venn diagram of differential genes obtained in the research studies using bioinformatics and evolutionary genomics (http://bioinformatics.psb.ugent.be/webtools/Venn/), and common differential genes were obtained.
- KEGG pathway analysis of common differential genes was made using DAVID (https://david.ncifcrf.gov/) and differential genes closely related to PD were screened [8].
- Protein-protein interaction of genes closely related to PD was figured out using STRING (https://string-db.org/), so as to make the protein-protein interaction closer and reduce the range of differential gene candidates. Confidence level ≥ 0.90 was set using STRING and protein-protein interactions, and differential gene candidates were obtained [9].
- Differential gene candidates were inputted into DGIdb dataset (http://dgidb.genome.wustl.edu/) so as to obtain the interrelation between drug and gene and drug candidates for treatment of PD. Relevant information of drug was obtained using PubChem dataset, the approval for clinic or clinical test was searched in ClinicalTrials dataset, and drug candidates were analyzed [10, 11].

## 3. Result

**Figure 2:**
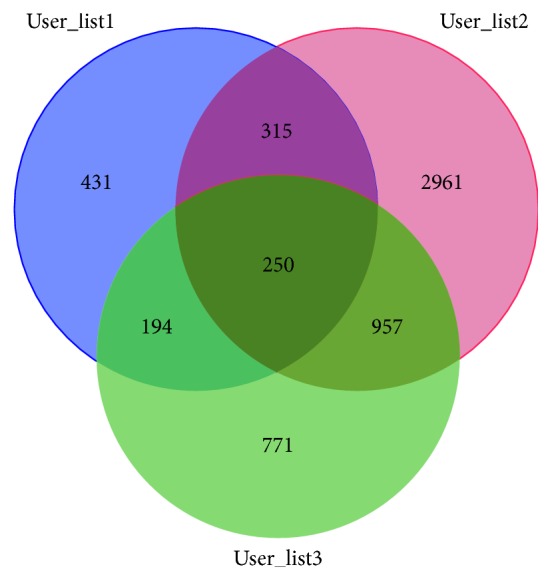
Venn diagram of 3 groups of differential genes. List 1, list 2, and list 3 were differential genes of chips GSE8397, GSE19587 and GSE8397, and GSE20333 respectively. 431, 2961, and 771 genes of 3 groups were identified by bioinformatics and evolutionary genomics, respectively, and 3 groups had 250 common differential genes.

One hundred sixty-three retrieved results were obtained from the retrieval of “PD (Parkinson's disease)” and “gene expression profiling” in GEO dataset, and based on strict screening, 3 of them conformed the screening requirements and can be applied to our research studies (Moran et al. [12] (chip number: GSE8397); Lewandowski et al. [13] (chip number: GSE19587); Edna et al. [14] (chip number: GSE20333)). 3 searches were analyzed using RStudio software, and 1191, 4484, and 2173 differential genes were obtained, respectively. The Venn diagram of 3 groups of differential genes was drawn using “bioinformatics and evolutionary genomics,” as shown in Figure 2, and 250 common differential genes were obtained.

**Figure 3:**
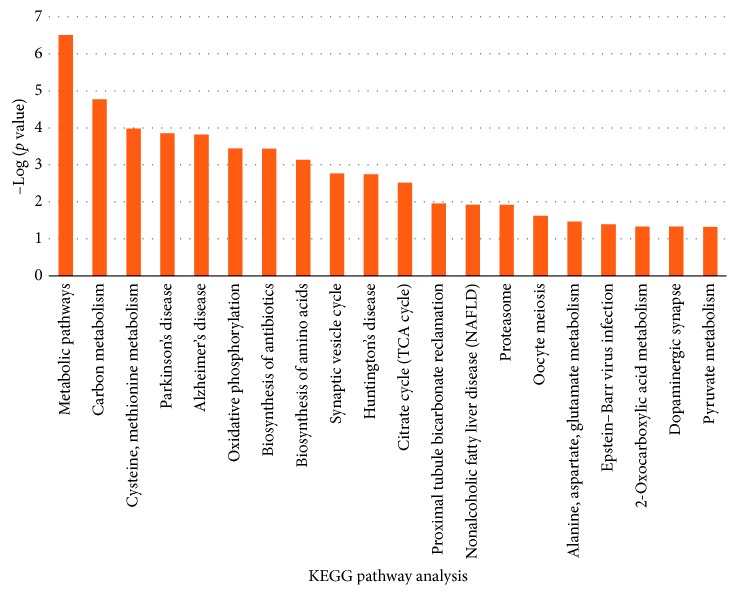
KEGG pathway analysis of 250 differential genes whose *P* value was less than 0.05.

KEGG pathway analysis was made for 250 differential genes using DAVID, and pathways whose *P* value was less than 0.05 were screened.  Figure 3 showed KEGG pathway analysis of 250 differential genes whose *P* value was less than 0.05. Metabolic pathways, carbon metabolisms, cysteine and methionine metabolism, and Parkinson's disease were 4 KEGG pathways with a smaller *P* value, and they contained 51, 12, 7, and 12 differential genes, respectively, as shown in Table 1. 82 differential genes of the aforesaid 4 pathways were further analyzed.

**Figure 4:**
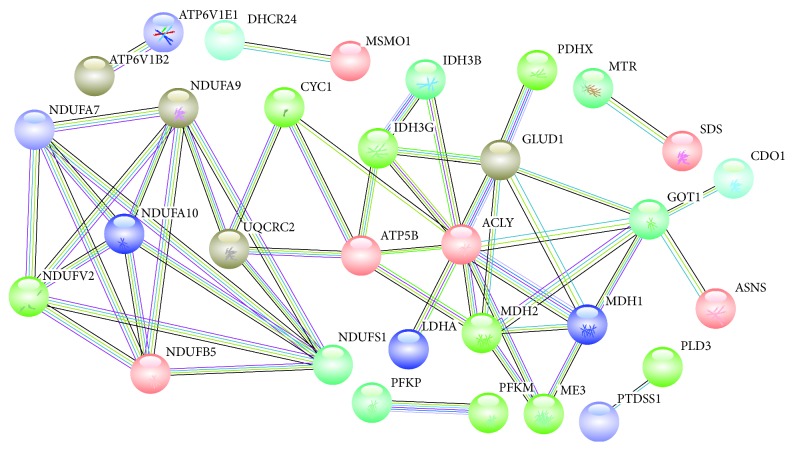
Thirty-one protein-protein correlations.

Protein-protein interaction of 82 differential genes of the aforesaid 4 pathways was obtained using STRING, and confidence level ≥ 0.90 was set in STRING so as to ensure proteins were closely related. As shown in Figure 4, 31 proteins were closely connected. It indicated that the drug had direct or indirect effect on 31 proteins when it acted on one or more proteins.

31 differential gene candidates were inputted into DGIdb dataset so as to obtain the drug interacting with the aforesaid genes, and 10 drug candidates were obtained through screening their biological characteristics and clinical application. As shown in Table 2, 10 drugs, such as metformin hydrochloride, were directly or indirectly correlated to differential gene candidates.

## 4. Conclusions

Drugs that may be used for prevention and treatment of PD were discovered through data mining and pathway analysis, which provided new ideas for drug R&D.

Based on the research result and information retrieval relating to drug candidates, 10 drugs such as metformin hydrochloride had certain therapeutical effect on PD, and melbine (DMBG) can be used for treatment of PD and type 2 diabetes patients.

## 5. Discussion

PD had a high morbidity, and drug therapy represented by dopaminergic drugs was a main treatment method for PD [15]. Nevertheless, anti-Parkinson's disease drugs mainly acted on dopamine metabolism and cholinergic metabolic pathways instead of the apoptosis mechanism of dopaminergic neuron, so it can neither inhibit the progress of the disease effectively nor cure the disease. Whereas traditional drugs synthesized based on physical tests and drug compounds featured long R&D cycle, high investment, high risk, and poor curative effect of clinical test, anti-Parkinson's disease drugs fell behind in drug R&D and could not accommodate to the rapid growth of PDs [16].

Data mining was an emerging research area in recent years that aimed to excavate potential and possible data pattern, internal relation, rule and development trend, etc. from unorganized data information, extract effective, novel, useful, understandable, and scattered valuable knowledge from text files and to make use of such knowledge for better information organization. Data mining of biomedical literature was construed as effective formation of research hypothesis, since it can reveal a new relationship between gene and pathogenesis. New evidences of adjustment of existing indications for drug therapy can be obtained through data mining combined with other bioinformatics tools [17–19] and reliable conclusions were drawn from drug R&D based on data mining.

In this research, macromining and microanalysis were combined innovatively, the direction of drug screening was determined, and targeting and high efficiency of drug mining were guaranteed based on big data analysis, bioinformatics analysis, and molecular pathology. 3 research studies were included in this research based on data mining of PD in GEO dataset. 250 differential genes were obtained from 3 groups of differential genes after gene crossover based on data mining of original gene and underwent KEGG analysis. KEGG pathway included Parkinson's disease and other pathways. 82 differential genes selected from 4 KEGG pathways with a smaller *P* value were further analyzed in order to ensure the reliability of differential gene candidates, drug-gene correlation of 31 differential gene candidates was analyzed, and finally 10 drug candidates were screened out.

Some potential therapeutic drugs for PD were discovered in our research studies, among which melbine (DMBG) was worth noting. Current research studies showed that mitochondrial function disorder, abnormal protein aggregation, neuroinflammation increase, and impaired cerebral glucose metabolism were common processes of insulin resistance, diabetes, and nervous system degeneration and have been identified as the key mechanism for the progress of PD and cognitive disorder [20]. Besides, it has been considered that melbine (DMBG) cannot be applied to treatment of type 2 diabetes through adjustment of glucose metabolism disorder, but it had obvious protective effect on the nerve cells of PD and other nervous system degenerations [21]. This research showed that metformin hydrochloride can be combined with NDUFA10, NDUFA7, NDUFA9, NDUFB5, NDUFS1, NDUFV2, and other acceptors and thus affect the mitochondrial respiratory chain. Information retrieval indicated that melbine (DMBG) had therapeutic effect on the animal model of PD, and epidemiological survey also indicated that it had effect on prevention and treatment of PD. Mark et al. carried out epidemiological survey on type 2 diabetes patients with PD and found that the probability of type 2 diabetes patients suffering PD was 2.2 times higher than normal people. However, melbine (DMBG) could control blood glucose so as to reduce the probability of type 2 diabetes patients suffering PD [22, 23]. Kang et al. discovered that melbine (DMBG) could mediate ATF2/CREB-PGC-1*α* pathway, induce proteomic change of metabolisms and mitochondria pathways in the substantia nigra, increase mitochondrial protein in the substantia nigra and the corpus striatum, protect dopaminergic neuron in the substantia nigra and the corpus striatum, and improve dyskinesia of PD [24]. Julia et al. discovered that TRAP1 could adjust the mitochondrial function of downstream PINK1 and HTRA2, malfunction of TRAP1 increased free NADH, mitochondria was produced, unfolded protein reaction and membrane potential of mitochondria were triggered, the sensitivity of mitochondria elimination and apoptosis decreased, and PD patients suffered TRAP1 malfunction, while metformin hydrochloride could adjust energy metabolism, produce mitochondria, recover the mitochondrial membrane potential, reverse mitochondrial mitochondrial function arising from TRAP1 mutation of PD, and provide new ideas for mitochondrial pathological change and treatment of PD [22, 25, 26]. Nevertheless, metformin hydrochloride had many untoward effects. Gastrointestinal reaction was the most common untoward effect, and other uncommon untoward effects included lactic acidosis, cutaneous anaphylaxis, hepatorenal damage, hypoglycemia, hematological damage, acute pancreatitis, neural abnormity, etc. [27, 28]. Therefore, metformin hydrochloride may have great clinical effect on treatment of PD, but we suggested using melbine (DMBG) for treatment of PD with type 2 diabetes and paying attention to the side effects.

Other than melbine (DMBG), other potential therapeutic drugs for PD were also discovered in this research. Research studies showed that PD patients lacked V12 but had higher HCY (homocysteine), so V12 could improve cognitive disorder and other symptoms of PD [29]. Clinical tests on vitamin B12 for treating cognitive disorder and other nonmotor symptoms of PD have been terminated [30]. In this research, hydroxocobalamin (namely vitamin B12) had mutual effect with MTR and affected transmethylation of methyl cobalamin to HCY (homocysteine) and consequently it gave play to neuroprotection. Folic acid was similar to hydroxocobalamin, both were the substrates of nerve regression and showed the potential to improve nonmotion functional disorder of PD. Methionine took part in many important physiological processes of human body, it has been rarely reported on PD and may have the potential to treat PD. Citric acid was an anticoagulant drug that took part in tricarboxylic acid cycle, affected the production of mitochondrial energy, and had a certain potential to treat PD [31]. L-glutamate interacted with ASNS, GLUD1, and GOT1. The interaction between dopamine and glutamic acid in the basal ganglion played an important part in the adjustment of motion and cognitive behavior, and functional disorder of dopaminergic-glutamic acid pathway was discovered in PD pathology [32–34]. Serine was an amino acid and research studies indicated that amino acid of D-serine could improve behavior and motor symptoms of PD [35, 36]. Moreover, niacinamide interacted with LDHA. Research studies showed that niacinamide protected dopaminergic neuron from neural degeneration induced by MPTP in the PD rat model, and it had protective effect on dopaminergic neuron [37, 38]. Niacinamide was a constituent part of coenzyme I and coenzyme II and became a coenzyme of many dehydrogenase. It has been approved to be used for prevention of endemic erythema and aniacinosis. Meanwhile, niacinamide could improve cognitive disorder of Alzheimer disease and has entered the second clinical test stage [39]. Pyridoxal phosphate, namely, vitamin B6, was a coenzyme of aminopherase and decarboxylase in the amino acid metabolism that could promote decarboxylation of glutamic acid. It has been discovered that oral administration of a moderate amount of vitamin B6 had preventive effect on PD [40, 41]. Furthermore, pyridoxal phosphate could relieve tardive dyskinesia of schizophrenia and schizoaffective disorder patients and has entered the second test stage [42]. Quercetin was an active component of many traditional Chinese medicines. It has been approved to be used for reducing the friability of blood corpuscle and had neuroprotective effect on dopaminergic neuron due to antioxygenation [43, 44].

This research still has some disadvantages. It is currently believed that Parkinson's disease is a neurodegenerative disease, caused by a combination of genetic factors, environmental factors, and nervous system aging, through oxidative stress, proteasome dysfunction, inflammation/immune response, mitochondrial dysfunction, calcium homeostasis, excitatory toxins, apoptosis, etc., leading to the degeneration and loss of substantia nigra dopaminergic neurons [45]. The susceptibility gene of Parkinson's disease is part of the pathogenic factor, so the efficacy of Parkinson's disease-based therapeutic drugs based on the Parkinson's disease susceptibility gene needs to be further evaluated. 3 research studies failed to identify the drugs for PD during the lifetime, so differential genes between PD group and the control group may be caused by other pathogenic factors. The drug candidates failed to further verify the potential therapeutical effect on PD. However, this research provided new ideas for R&D of PD drugs and potentially safe drugs for treatment of PD.

In short, drugs that may be used for prevention and treatment of PD can be obtained from data mining and bioinformatics analysis, which provided new ideas for drug R&D and research studies on “new application of conventional drugs.” Metformin hydrochloride and other drugs had great clinical effect on treatment of PD, so melbine (DMBG) can be used for treatment of PD and type 2 diabetes patients, which can be proved by more clinical tests.

## Figures and Tables

**Table 1:** Differential genes of 4 DAVID KEGG pathways with a smaller *P* value.

KEGG pathway	Gene number	*P* value	Genes
Metabolic pathways	51	3.08*E* − 07	UQCRC2, SGSH, LDHA, IMPA1, HMGCR, ATP5B, CYC1, GSS, ALAS1, IDH3G, GOT1, PIGB, PTDSS1, NDUFS1, DHCR24, PLD3, CMAS, PFKP, PFKM, DGUOK, CDO1, NDUFA10, SDS, OAT, MDH2, MDH1, ME3, NDUFB5, SORD, GLUD1, UROS, ASNS, ATP6V1B2, ALDH1A1, ENO2, PAFAH1B1, PTS, PDHX, MTMR4, POLR3F, MSMO1, MOCS2, NDUFA9, NDUFA7, IDH3B, ACLY, ATP6V1E1, NDUFV2, MTR, QPRT, HIBCH
Carbon metabolism	12	1.70*E* − 0.5	ME3, GOT1, IDH3G, SDS, GLUD1, ENO2, PFKP, IDH3B, HIBCH, PFKM, MDH2, MDH1
Cysteine and methionine metabolism	7	1.05*E* − 0.4	LDHA, GOT1, SDS, MTR, CDO1, MDH2, MDH1
Parkinson's disease	12	1.40*E* − 0.4	UQCRC2, NDUFB5, NDUFA9, ATP5B, CYC1, NDUFA7, NDUFV2, UCHL1, SLC18A2, NDUFA10, VDAC3, NDUFS1

**Table 2:** Thirty-one drug candidates for differential gene prediction (drug-gene connection table).

Drug	Gene	Interaction types	Approved?	Administration	Approved use	PubMed ID
Citric acid	MDH2	N/A	Yes	Oral administration	Anticoagulation	10592235
Folic acid	MTR	N/A	yes	Oral administration	Trophic nerve	
Hydroxocobalamin	MTR	Cofactor	Yes	Oral administration/intravenous drip	Neuroprotection	18565, 1744096, 7599160, 3812589, 9587031
L-glutamate	ASNS, GLUD1, GOT1	N/A	Phase 3	Oral administration	Neuroprotection	17139284, 17016423, 8288265, 17139284, 17016423, 17444813
Metformin hydrochloride	NDUFA10, NDUFA7, NDUFA9, NDUFB5, NDUFS1, NDUFV2	Inhibitor	Yes	Oral administration	Hypoglycemic effect	
Methionine	MTR	Product	Yes	Oral administration	Liver protection	17222188, 16618098, 17615995, 16788729, 17052662
Niacinamide	LDHA	N/A	Yes	Oral administration	Cardiac disease, cognitive disorder	10592235, 17139284, 17016423
Pyridoxal phosphate	GOT1, SDS	Activator	Phase 2	Oral administration	Dyskinesia	11340119, 16925884, 12167474, 11888303, 11752352, 14596599, 15155761, 14646100, 16580895, 15689518
Quercetin	ATP5B	N/A	Phase 1	Oral administration	Pain	10592235
Serine	SDS	N/A	Phase 2	Oral administration	Cognitive improvement	4377655, 14688104, 17139284, 17016423, 500557

## Data Availability

All data reported in this manuscript are included within the article. Raw data, if required, are available on request.
